# Differential Immune Responses to *Segniliparus rotundus* and *Segniliparus rugosus* Infection and Analysis of Their Comparative Virulence Profiles

**DOI:** 10.1371/journal.pone.0059646

**Published:** 2013-03-29

**Authors:** Jong-Seok Kim, Woo Sik Kim, Keehoon Lee, Choul-Jae Won, Jin Man Kim, Seok-Yong Eum, Won-Jung Koh, Sung Jae Shin

**Affiliations:** 1 Department of Microbiology and Institute for Immunology and Immunological Diseases, Yonsei University College of Medicine, Seoul, South Korea; 2 Department of Pathology, College of Medicine, Chungnam National University, Daejeon, South Korea; 3 Division of Immunopathology and Cellular Immunology, International Tuberculosis Research Center, Changwon, South Korea; 4 Division of Pulmonary and Critical Care Medicine, Department of Medicine, Samsung Medical Center, Sungkyunkwan University School of Medicine, Seoul, South Korea; Cornell University, United States of America

## Abstract

Two closely related bacterial species, *Segniliparus rotundus* and *Segniliparus rugosus*, have emerged as important human pathogens, but little is known about the immune responses they elicit or their comparative pathophysiologies. To determine the virulence and immune responses of the two species, we compared their abilities to grow in phagocytic and non-phagocytic cells. Both species maintained non-replicating states within A549 epithelial cells. *S. rugosus* persisted longer and multiplied more rapidly inside murine bone marrow-derived macrophages (BMDMs), induced more pro-inflammatory cytokines, and induced higher levels of macrophage necrosis. Activation of BMDMs by both species was mediated by toll-like receptor 2 (TLR2), followed by mitogen-activated protein kinases (MAPK) and nuclear factor κB (NF-κB) signaling pathways, indicating a critical role for TLR2 in *Segniliparus*-induced macrophage activation. *S. rugosus* triggered faster and stronger activation of MAPK signaling and IκB degradation, indicating that *S. rugosus* induces more pro-inflammatory cytokines than *S. rotundus.* Multifocal granulomatous inflammations in the liver and lung were observed in mice infected with *S. rugosus*, but *S. rotundus* was rapidly cleared from all organs tested within 15 days post-infection. Furthermore, *S. rugosus* induced faster infiltration of innate immune cells such as neutrophils and macrophages to the lung than *S. rotundus*. Our results suggest that *S. rugosus* is more virulent and induces a stronger immune response than *S. rotundus*.

## Introduction

*Segniliparus rugosus* and *S. rotundus* are the only two species belonging to the genus *Segniliparus*, first identified in 2005, which is the sole genus in the family *Segniliparaceae* [1]. This family is of great interest because it has novel and extremely long carbon-chain fatty acids (mycolic acids) embedded in the cell wall [1]. These molecules distinguish the *Segniliparus* spp. from those of *Rhodococcus* and *Mycobacterium* in terms of genetic, physiological, and chemical properties [1]. The type strains of *S. rugosus* and *S. rotundus* are CDC 945^T^ (ATCC number: BAA-974^T^; CIP number: 108380^T^) and CDC 1076^T^ (ATCC number: BAA-972^T^; CIP number: 108378^T^), respectively [1]. Both type strains of the genus *Segniliparus* were isolated from human sputum originally suspected as containing nontuberculous mycobacteria because the cell walls contained mycolic acids and the rod-shaped bacilli had positive acid–alcohol-fast staining [2], [3], [4]. The characterization study demonstrated that they shared some phenotypic characteristics with rapidly growing mycobacteria, but most rapidly growing mycobacteria stain weakly acid-fast [5]. The *Segniliparus* spp. exhibited surprisingly intense acid-fast staining, and this suggested that the mycolate structures in these unusual bacteria may exhibit novel properties.

Accurate identification of bacteria is important for evaluating the clinical implications of emerging pathogens in respiratory infections. *Segniliparaceae* may be confused with nonchromogenic, rapidly growing mycobacteria in microscopic examination due to the acid-fast staining properties of these species. Clinicians and physicians should be aware that acid-fast bacteria other than *Mycobacterium* spp. exist in respiratory infections, and further studies are needed to investigate the significance and clinical importance of the *Segniliparus* spp.

*S. rugosus* has recently been reported in patients with cystic fibrosis in the United States and Australia, and a case of *S. rotundus* pneumonia in a patient with non-cystic fibrosis bronchiectasis has been reported in Korea [2], [3], [6], [7]. These cases suggest that *Segniliparus* spp. may be emerging respiratory pathogens that can cause pneumonia in patients with bronchiectasis. Although few studies of *Segniliparus* infection have been published, and reliable information about their pathogenesis is limited, more cases of *S. rugosus* lung disease, including in animals, have been reported than from *S*. *rotundus*. In addition, *in vitro* susceptibility testing of both type strains found that the *S. rotundus* reference strain and isolate were susceptible to several oral antibiotics, including clarithromycin, ciprofloxacin, moxifloxacin, and sulfamethoxazole, but the *S. rugosus* reference strain was highly resistant to these antibiotics [2], [3], [7]. Thus, *S. rugosus* may be more pathogenic than *S. rotundus* in terms of antibiotic resistance and disease frequency.

In recognition of the medical importance of the *Segniliparus* spp., the genomes of both species have recently been sequenced [8], [9]. However, very little information regarding their relative pathogenicities or the host immune responses they elicit is available in this sequencing data. Thus, experiments aimed at understanding host molecular immunity to newly identified pathogens and their pathogenesis are critical for the development of effective strategies to control any diseases that they may cause.

Many pathogens trigger signaling pathways through molecules such as mitogen-activated protein kinase (MAPK) and nuclear factor κB (NF-κB) that are involved in the cytokine response and inflammation [10], [11]. These responses are initiated through pattern recognition receptors (PRRs) that recognize and respond to pathogen-associated molecular patterns (PAMPs) [10]. Upon PAMP-PRR interactions, the appropriate immune responses to the pathogens can be initiated to help maintain well-regulated immunologic homeostasis [10]. Toll-like receptors (TLRs) are the most well known PRRs and play a crucial role in the activation of the cellular immune response against many pathogenic bacteria [12]. Activation of signaling through Toll/interleukin-1 receptor (TIR) domains results in recruitment of the adaptor molecules MyD88 and/or TIR-domain-containing adapter-inducing interferon-β (TRIF), and this ultimately leads to activation of MAPKs and NF-κB [10], [12]. Delineating the functions of these molecules is thus important for understanding how host resistance is induced, maintained, and regulated.

Very little information is available on the early stages of infection that initiate the immune response against *Segniliparus* infections or the later stages that sustain and regulate this response. In addition, host immune responses against many pathogenic bacterial infections differ, even in the same species, depending on their differences in virulence. In the present study, we comparatively investigated the phenotypic differences in the pathogenesis and immune responses of *S*. *rugosus* and *S*. *rotundus* infections using murine bone marrow-derived macrophages and *in vivo* infection models.

## Materials and Methods

### Reagents and Antibodies

Recombinant mouse macrophage colony stimulating factor (M-CSF) and the phycoerythrin (PE)-annexin V/7-AAD kit were purchased from R&D Systems (Minneapolis, MN, USA). Anti-phosphorylated ERK1/2 mAb, anti-ERK1/2 polyclonal Ab, anti-phosphorylated p38 mAb, anti-p38 polyclonal Ab, anti-phosphorylated IκB-α mAb, anti-IκB-α mAb were obtained from Santa Cruz Biotechnology, Inc. (Santa Cruz, CA, USA). HRP-conjugated anti-mouse IgG Ab and HRP-conjugated anti-rabbit Ab were obtained from Calbiochem (San Diego, CA, USA), and anti-β-actin mAb (AC-15) was purchased from Sigma. ELISA kits for TNF-α, IL-6, IL-4, IL-17 and IFN-γ were obtained from eBioscience (San Diego, CA, USA) and IL-12p70 and IL-10 ELISA kits were obtained from BD Biosciences (San Diego, CA, USA). FITC-conjugated mAb for CD11b, PE-cy7-conjugated mAb for CD11c, Alexa700-conjugated mAb for Gr-1 and APC-conjugated mAb for F4/80 were purchased from eBioscience (San Diego, CA, USA). Monoclonal biotinylated rat anti-mouse IgG1 and IgG_2a_ were purchased from Invitrogen (Carlsbad, CA, USA).

### Bacteria Culture

The reference strains of *S. rugosus* CIP 108380^T^ and *S. rotundus* CIP 108378^T^ obtained from Institute Pasteur (Paris, France) were used in the present study [1]. These strains were initially cultured in 7H9 broth (Becton Dickinson, Franklin Lakes, NJ, USA) supplemented with oleic acid-albumin-dextrose-catalase (OADC; Becton Dickinson, Sparks, MD, USA) for 10 days at 37°C. Single-cell suspensions of each strain were prepared as previously described with slight modifications. Seed lots of each strain were kept in small aliquots at −80°C until use. Ten-fold serial dilutions from seed lots of each strain were plated on Middlebrook 7H10 agar (Becton Dickinson, Franklin Lakes, NJ, USA) to quantify the number of organisms per milliliter.

### Ethics Statement

All animal experiments were performed in accordance with the Korean Food and Drug Administration (KFDA) guidelines. The experimental protocols used in this study were reviewed and approved by the Ethics Committee and Institutional Animal Care and Use Committee (Permit Number: 2012-0072-2) of the Laboratory Animal Research Center at Yonsei University College of Medicine (Seoul, Korea).

### Mouse

Specific pathogen-free female C57BL/6 mice at 5–6 weeks of age were purchased from Japan SLC, Inc. (Shijuoka, Japan) and maintained under barrier conditions in a BL-3 biohazard animal facility at the University Medical Research Center. The animals were fed a sterile commercial mouse diet and provided with water *ad libitum*.

### Generation of Bone-marrow Derived Macrophage

Murine bone marrow-derived macrophages (BMDMs) were differentiated for 6 days in M-CSF-containing media, as described previously. Briefly, bone marrow cells from the femur and tibia were cultured in Dulbecco’s modified Eagle’s medium (DMEM; HyClone, Logan, UT, USA) containing 2 mM l-glutamine, 100 U/ml penicillin, 100 µg/ml streptomycin, 10% fetal bovine serum (FBS; Invitrogen, Carlsbad, CA, USA), and 20 ng/mL of recombinant macrophage colony-stimulating factor (M-CSF) (R&D Systems, Minneapolis, MN, USA) at 37°C in the presence of 5% CO_2_. After 6 days, non-adherent cells were removed, and the differentiated macrophages were incubated in antibiotic-free DMEM until use.

### Bacteria Staining, SEM and Confocal Microscopy

The size of the cells was determined by scanning electron microscopy from specimens fixed with 5% glutaraldehyde in 0.1 M cacodylate buffer for 4 h at room temperature. Suspensions were filtered with 0.1 µm polycarbonate filters (Porectics Corporation, Livermore, CA, USA), dehydrated in ethanol and immersed in hexamethyldisilazane overnight at room temperature. Preparations were sputter-coated with 30 nm gold on aluminium mounts and observed with a Philips XL 30 Environmental scanning electron microscope (FEI Company, Portland, OR, USA).

To investigate whether *S. rugosus* and *S. rotundus* are intracellular bacteria, we used carboxyfluorescein diacetate succinimidyl ester (CFSE; Invitrogen, Carlsbad, CA, USA) for staining of bacteria as previously described [14]. Briefly, 1×10^8^ cells/ml of bacteria were washed once in phosphate buffered saline (PBS) and diluted in 500 µl of PBS. PBS (500 µl) containing 10 µM CFSE was added to the bacterial suspension and incubated for 10 min at room temperature. The bacterial suspension was washed twice in PBS supplemented with 5% FBS. Infection was carried out at a bacterium/macrophage ratio of approximately one for confocal analysis. After 4 h incubation, cells were washed three time in PBS, fixed in 3.7% formaldehyde in PBS for 15 min at room temperature, permeabilized in 0.05% Triton X-100, and stained for actin with Phalloidin-Texas Red (Invitrogen, Carlsbad, CA, USA) [15]. After washing, specimens were mounted in Fluoroshiled™ with DAPI (Sigma, St Louis, MO, USA). CFSE-coupled bacteria, actin, nucleic acid were observed using an Olympus FV-1000 fluorescence microscope (Dulles, VA, USA).

### Growth of *S. rugosus* and *S. rotundus* in BMDMs and A549 Cells

BMDMs and A549 cells were plated in 24-well culture plates at a density of 1×10^5^ and 5×10^4^ cells per well, respectively. After 24 h, BMDMs and A549 cells were infected at a multiplicity of infection [16] of 1 for 4 h at 37°C. After 4 h, the cells were washed 3 times with prewarmed serum-free media to remove all extracellular bacteria and further cultivated in fresh complete medium for 5 days. At days 0, 1, 2, 3, 4 and 5, the culture supernatants were aspirated and cells were lysed by incubation with distilled water containing 0.01% Triton X-100 (Sigma, St Louis, MO, USA), and the lysates were plated in ten-fold serial dilutions onto 7H10 agar (Becton Dickinson, Franklin Lakes, NJ, USA ) to quantify the number of viable bacteria. Colonies were counted after 1 weeks of incubation at 37°C. The resultant values were reported as the mean log_10_CFU ± standard deviation (SD) per 1×10^5^ cells. To investigate whether *S. rugosus* and *S. rotundus* present the similar infection kinetics during the first 4 h, BMDMs were infected at a multiplicity of infection of 10 per 1×10^5^ of cells for 10, 30, 60, 120, 180 and 240 min at 37°C. After incubation, the cells were washed 3 times with prewarmed serum-free media to remove extracellular bacteria. Cells were lysed with distilled water containing 0.01% Triton X-100 and the lysates were plated in ten-fold serial dilutions onto 7H10 agar to quantify the number of infected bacteria. Colonies were counted after 1 week of incubation at 37°C. The resultant values were reported as the mean log_10_CFU ± SD per 1×10^5^ cells.

### Flow Cytometric Analysis

For the determination of cell death type followed by *S. rugosus* and *S. rotundus* infection, cells were infected at a MOI of 10 bacteria. After 20 h, cells were harvested, washed with ice-cold PBS, and resuspended in 1 ml of Annexin V binding buffer (10 mM HEPES [pH 7.4], 140 mM NaCl, 2.5 mM CaCl_2_). Next, 1×10^5^ cells were stained with 5 µl of Annexin V-PE and 5 µg/ml of **7**-AAD in 100 µL of Annexin V binding buffer at 4°C. After 20 min, 400 µL of binding buffer was added to each tube and the samples were analyzed using a FACS Canto flow cytometer (BD Biosciences, San Jose, CA, USA). The cells were assigned to one of four states: alive, annexin V-negative, and 7-AAD-negative; early apoptotic, annexin V-positive, and 7-AAD-negative; late apoptotic, annexin V-positive, and 7-AAD-positive; or necrotic, annexin V-negative, and 7-AAD-positive.

For flowcytometric analysis of infected cells, 5×10^5^ cells of BMDM or A549 in 6 cm culture dishes were infected at a MOI of 1 or 10 CFSE-labeled bacteria/cells. After 4 h of infection, the cells were washed 3 times with prewarmed PBS to remove extracellular bacteria and cells were resuspended by 5 min trypsin treatment. The infected cells were washed 3 times with ice-cold PBS and resuspended in 1 mL PBS. To eliminate signals from extracellular bacteria, trypan blue solution (0.4%; Sigma, St Louis, MO, USA) was treated at a final concentration of 0.2% for 10 min. Samples were analyzed by FACS Canto flow cytometer (BD Biosciences, San Jose, CA, USA).

### Lactate Dehydrogenase (LDH) Release Assay

The release of LDH from BMDMs (1×10^5^ cells/48-well culture plate) infected with bacteria was measured using a Cytotoxicity Detection Kit^PLUS^ (Roche, Indianapolis, IN, USA) according to the manufacturer’s protocol. Relative cytotoxicity (%) was calculated as the percentage of LDH released from the bacteria-infected cells divided by the maximum LDH released.

### Cytokine Measurement by Enzyme-linked Immunosorbent Assay (ELISA)

Supernatants from the bacteria-infected BMDMs (1×10^5^ cells/48-well culture plate) and non-infected BMDMs were collected at 24 h post infection, sterile-filtered, and then stored at −80°C until use. Serum from the infected mice were collected at 2, 5, 15 days after infection, then stored at −80°C until use. The levels of tumor necrosis factor (TNF)-α, interleukin (IL)-6, IL-12p70, IL-10, IL-17 and IL-4 were determined by ELISA using commercial kits following the manufacturer’s instructions.

### Levels of Antibody Responses, IFN-γ, IL-4, and IL-17 Production in Mice Following Infection with *S. rugosus* and *S. rotundus*

For the determination of serum level of IgG subclasses, serum samples were obtained from each mouse, and the IgG1 and IgG_2a_ were quantified. Briefly, 96-well microtiter plates (MaxiSorp; Nunc, Denmark) were coated with 50 µL antigen preparations diluted in carbonate buffer (pH 9.6) at a final concentration of 1 µg/ml for each bacterial lysate antigens. After incubation, unbound antigen was removed from the plates by washing them six times in phosphate-buffered saline containing 0.05% Tween 20 (wash buffer). One hundred microliter of serum (1∶100 dilution in wash buffer) were added to separate wells for each antigen, incubated for 1 h at 37°C, and then washed a further six times. Monoclonal biotinylated rat anti-mouse IgG1 (100 µL/well, 1∶500 dilution) or IgG_2a_ (100 µL/well, 1∶500 dilution) were then added and incubated for 1 h at 37°C, and unbound antibody was removed by washing six times. Antibody binding was visualized using streptavidin conjugated to HRP (Serotec, Oxford, UK) and an 3,3′,5,5′-tetramethylbenzidine (TMB; Sigma, Saint-Louis, MO) substrate system. The reaction was stopped by the addition of H_2_SO_4_, and the Optical densities (OD) were read at 450 nm. Data represent the mean value from duplicate determinations of individual mice. Control naive mouse sera gave OD less or equal to zero.

For the determination of cell-mediated immune response, splenocytes were obtained from mice of each group, and the IFN-γ, IL-4, and IL-17 productions were quantified after treatment with 10 µg/mL of cellular extract (CE) antigens from *S. rugosus* and *S. rotundus*. To prepare CE antigens from *S. rugosus* and *S. rotundus* were cultivated in modified Watson-Reid medium (mWR, pH 6.0) at 37°C for 2 weeks, as previously described [17]. Mice were euthanized with CO_2_ and splenocytes were obtained by preparing single-cell suspensions from spleen tissue dispersed with sterilized glass slides. Erythrocytes were lysed in a solution containing 155 mM ammonium chloride and 10 mM potassium bicarbonate buffer, and thoroughly washed. 2×10^5^ cells of splenocyte were stimulated with *S. rugosus* and *S. rotundus* CE antigens in 48-well tissue culture plates for 24, 48 and 72 h, culture supernatants collected, filtered through 0.45 um membrane, and frozen at −80°C until use. The levels of IFN-γ, IL-4, and IL-17 in culture supernatants were determined by ELISA using commercial kits following the manufacturer’s instructions.

### Western Blot Analysis

After infected with bacteria, adherent cells were washed twice with PBS, gently scraped from dishes, centrifuged, lysed in ice-cold lysis buffer (50 mM Tris-HCl pH 7.4, 0.8 M NaCl, 5 mM MgCl_2_, 0.5% NP-40, and protease inhibitor cocktail (Roche, Mannheim, Germany), and cleared by microcentrifugation. Equal amounts of protein were subjected to electrophoresis on 8–15% polyacrylamide gels containing SDS under reducing conditions. Separated proteins were electroblotted onto nitrocellulose membranes, and blots were incubated with 5% nonfat dry milk (wt/vol) for 1 hr and then washed in Tris-buffered saline containing 0.1% Tween 20 (TBS/T). Membranes were then incubated with primary antibody, and antibody binding was detected using the appropriate secondary antibody coupled with horseradish peroxidase (HRP), as described by the manufacturer. Enhanced chemiluminescence was used to detect relevant proteins, again by following the manufacturer’s instructions (Amersham, Buckinghamshire, UK).

### *In vivo* Infection

Specific pathogen-free female C57BL/6 (H-2K^b^ and I-A^b^) at 6 weeks of age were purchased from the Jackson Laboratory (Bar Harbor, ME, USA) and maintained under barrier conditions in a BL-3 biohazard animal facility at Yonsei University Medical Research Center according to the regulations of the Institutional Animal Care and Use Committee, Yonsei University Health System.

Bacterial survival and histopathology changed by intravenous infection with *S. rugosus* and *S. rotundus* were analyzed and compared. Briefly, two groups of mice (*n* = 30 per group) were infected intravenously with *S. rugosus* and *S. rotundus* by delivering 100 µl of the bacterial suspension containing 10^7^ CFU under anesthetic (ketamine and xylazine; Sigma, Saint-Louis, MO, USA). Five to six mice per group were euthanized at 2, 5, 15 and 28 days post-infection, and their lungs, spleens and livers were collected for histopathological and bacteriological examinations. The number of viable bacteria in the lung, spleen and liver was determined by plating serial dilutions of the organ homogenates onto Middlebrook 7H10 agar (Becton Dickinson, Franklin Lakes, NJ, USA). Colonies were counted after 1 weeks of incubation at 37°C. The resultant values were reported as the mean log_10_CFU ± SD per gram of lung tissue.

Tissue samples collected for histopathology were preserved in 10% neutral-buffered formalin, embedded in paraffin, cut into 4-5-µm sections, and stained with hematoxylin and eosin. Each specialized pathologist examined the tissue sections of liver and lung in a blinded manner, respectively. The inflammatory responses were ranked using a score of 0 to 4 based on lesion size and number per field. Tissues with more than three inflammation sites containing multiple and large-sized lesions were given a score of 4.

Bronchoalveolar lavage [18] samples were harvested by infusing 1 mL of ice cold PBS through a 25-gauge blund needle into the lungs via the trachea, followed by gentle aspiration of this fluid into a syringe at 2, 5 and 15 days post-infection [19]. BAL fluid and BAL cells were separated by centrifugation at 13,000×*g* for 10 min at 4°C. The supernatants were carefully collected for ELISA, and the cell pellets were collected for flow cytometric analysis. Murine IFN-γ, TNF-α, IL-12p70 and IL-10 were analyzed in BAL samples via ELISA (eBioscience, San Diego, CA), according to the manufacturer’s instructions. BAL cells were washed with ice-cold flow cytometry buffer (2% (vol/vol) bovine serum albumin and 2 mM EDTA in PBS, pH 7.5), then incubated with each antibody for 15 minutes and washed twice with flow cytometry buffer. Data were acquired on the FACSCanto II flow cytometer (BD Biosciences, San Diego, CA, USA) and analyzed with the FlowJo (Tree Star Inc., Ashland, OR, USA).

### Statistical Analysis

All experiments were repeated at least 3 times with consistent results. The levels of significance for comparison between samples were determined by Tukey’s multiple comparison test distribution using statistical software (GraphPad Prism Software, version 4.03; GraphPad Software, San Diego, CA). The data in the graphs are expressed as the mean ± SD. Each value of *p<0.05, **p<0.01 or ***p<0.001 was considered to be statistically significant.

## Results

### Morphological Study and Intracellular Growth Pattern of the *Segniliparus* spp. in Macrophages

**Figure 1:**
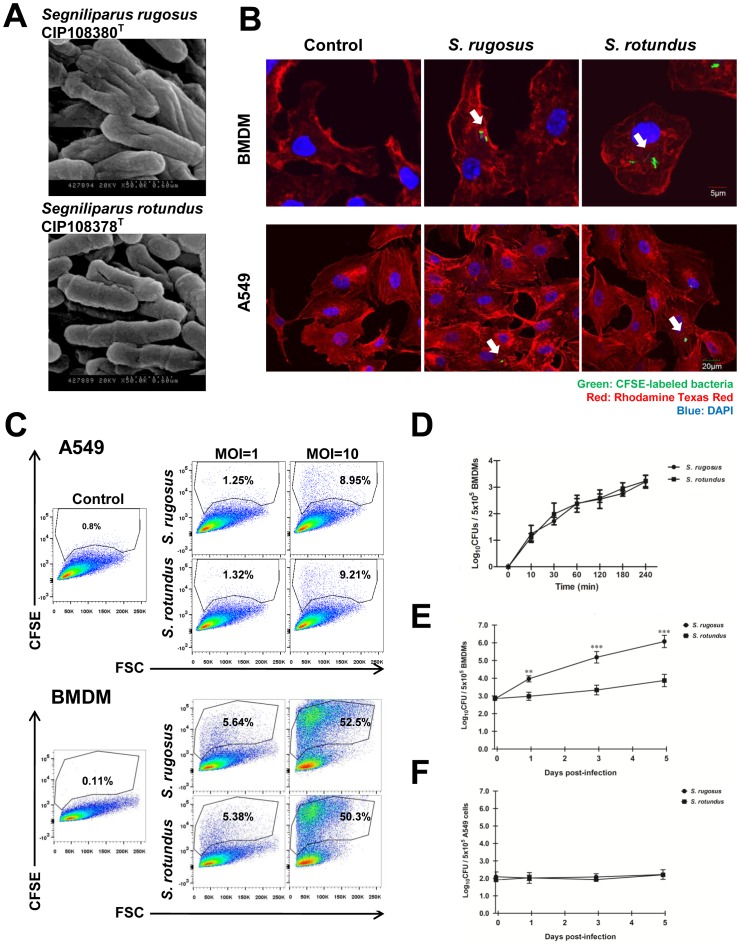
*S. rugosus* and *S. rotundus* are intracellular bacteria. A) Scanning electron microscope images of *S. rugosus* and *S. rotundus*. B) Intracellular staining of CFSE-coupled *S. rugosus* or *S. rotundus* in BMDMs (Scale bar, 5 µm) or A549 cells (Scale bar, 20 µm). The cortical F-actin was stained using Phalloidin-Texas Red. C) Flowcytometric quantitation of *S. rugosus* and *S. rotundus* in BMDMs and A549 epithelial cells. BMDMs and A549 cells were infected with the CFSE-coupled bacteria at a MOI of 1 or 10 for 4 h. Non-internalized bacteria were washed off three times and added 0.2% trypan blue. D) Infection kinetics of *S. rugosus* and *S. rotundus* in BMDMs during early 4 h post-infection. BMDMs (1×10^5^) were infected with *S. rugosus* and *S. rotundus* at a MOI of 10. At each indicated time-point, cells were washed three times and lysed with 0.05% Triton X-100 containing distilled water to release intracellular bacteria. This experiment was repeated three times with similar results. E, F) Growth profiles of *S. rugosus* and *S. rotundus* within A549 cells and BMDMs, respectively. BMDMs and A549 cells were infected with a MOI of 1 bacteria per cell. Non-internalized bacteria were washed off after 4 h. The number of bacterial colony forming units (CFU) was determined at the indicated time point after removing the supernatant. This experiment was repeated three times with similar results (**p<0.01, or ***p<0.001).

As previously reported, the cell morphotypes *S. rugosus* CIP 108380^T^ and *S. rotundus* CIP 108378^T^ were compared by scanning electron microscopy [1], [9]. The *S. rugosus* cells are rod-shaped, have irregular and rough surfaces, and tend to aggregate. The *S. rotundus* cells have a relatively round and smooth phenotype (Fig. 1A).

The abilities to replicate and to survive within host cells after infection are critical determinants of virulence for intracellular pathogens [20]. To examine whether *S*. *rotundus* and *S*. *rugosus* are selectively able to infect and replicate in phagocytic or non-phagocytic cells, bone marrow-derived macrophages (BMDMs) and A549 epithelial cells were infected with *S*. *rotundus* and *S*. *rugosus* marked with CFSE with a multiplicity of infection [16] of 1 bacterium per cell. The infection rates into macrophages and A549 cells did not differed significantly between *S*. *rotundus* and *S*. *rugosus* while both species infected macrophages greater than A549 cells (Fig. 1B and 1C). There was no significant difference in infection kinetics between the two species (Fig. 1D). Both species maintained non-replicating states within A549 epithelial cells at a MOI of 1 for 5 days post-infection while macrophages infected with *S. rugosus* had significantly higher rates of intracellular multiplication during the first 5 days post-infection than did cells infected with *S. rotundus* (*p*<0.001) (Fig. 1E and 1F). Thus, we concluded that *S. rugosus* is more virulent than *S. rotundus* in terms of its ability to survive and multiply inside macrophages.

### *S. rugosus* Induces High Levels of Cytokine Production in BMDM

**Figure 2:**
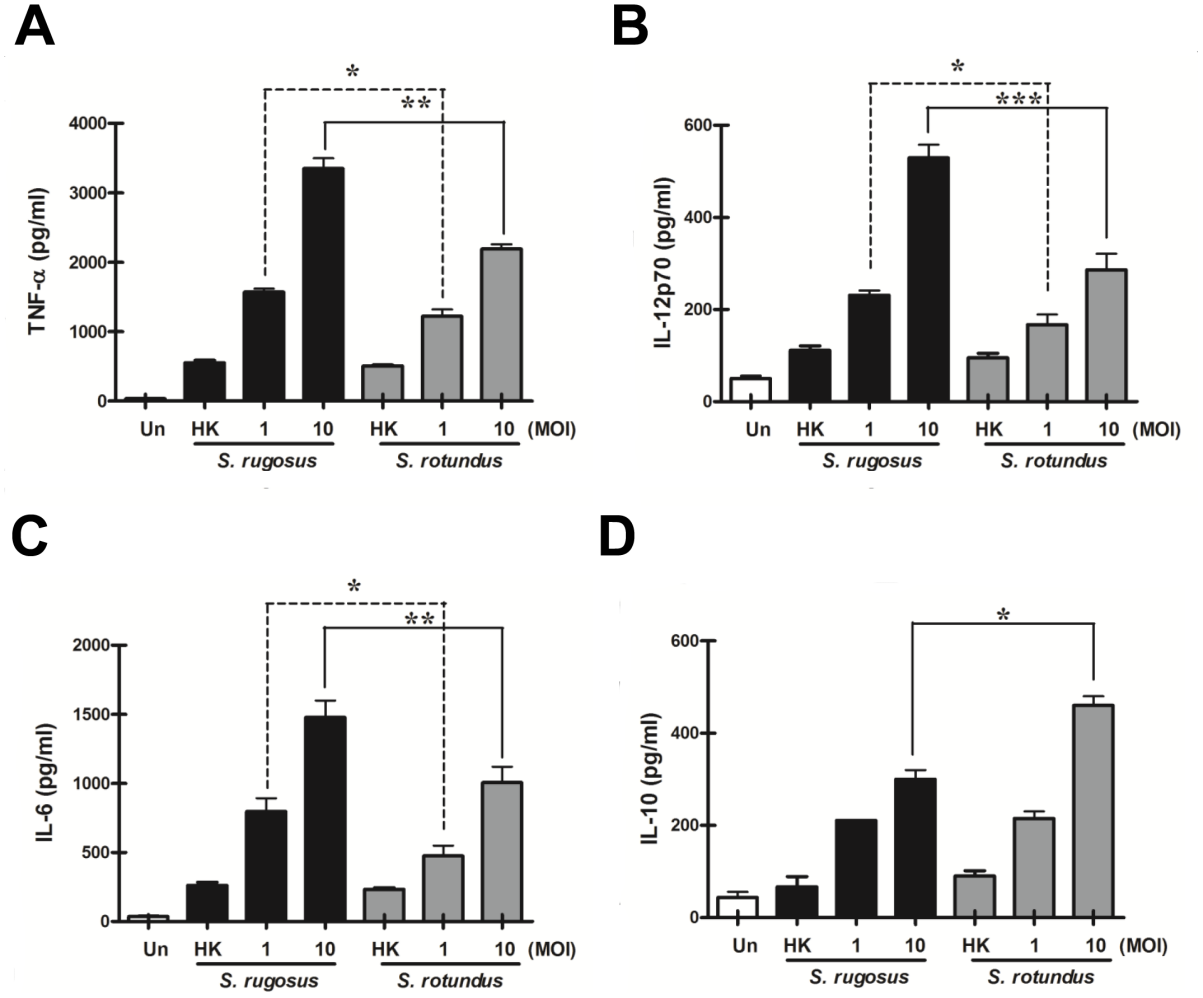
Comparison of inflammatory cytokine production by BMDMs infected with *S. rugosus* and *S. rotundus*. BMDMs were infected with *S. rugosus* or *S. rotundus* at a MOI of 1 or 10 for 24 h. Results represent mean pg/mL ± SD of all experiments performed. Supernatants were collected and the levels of TNF-*α* (A), IL-12p70 (B), IL-6 (C), and IL-10 (D) were determined by enzyme-linked immunosorbent assay. This experiment was repeated three times with similar results (*p<0.05, **p<0.01, or ***p<0.001). Un: uninfected; HK: Heat-killed bacteria (MOI = 10).

The ability to induce pro-inflammatory cytokines such as TNF-α, along with the ability to multiply rapidly in macrophages during intracellular bacterial infection, is an important indicator of virulence [18], [21]. We compared the abilities of two species to stimulate the release of cytokines from BMDMs at a MOI of 1 or 10 at 24 h post-infection. Overall, *S. rugosus* elicited significantly higher levels of the pro-inflammatory cytokines TNF-α, IL-12p70, and IL-6 than *S. rotundus* and in a MOI-dependent manner (Fig. 2A–C). Unlike the other cytokines, IL-10 was produced in comparable amounts by macrophages infected with both species at a MOI of 1, but macrophages infected with *S. rotundus* at a MOI of 10 produced significantly more IL-10 than those infected with *S. rugosus* at the same MOI (Fig. 2D). Thus, our results showed that the immune responses of macrophages differed in response upon infection by *S. rugosus* or *S. rotundus* by inducing different type of cytokines. Heat-killed bacteria induced lower levels of these cytokines and there were no significant difference between species (Fig. 2).

### Infection with *S. rugosus* Induces More Cell Death in BMDMs

**Figure 3:**
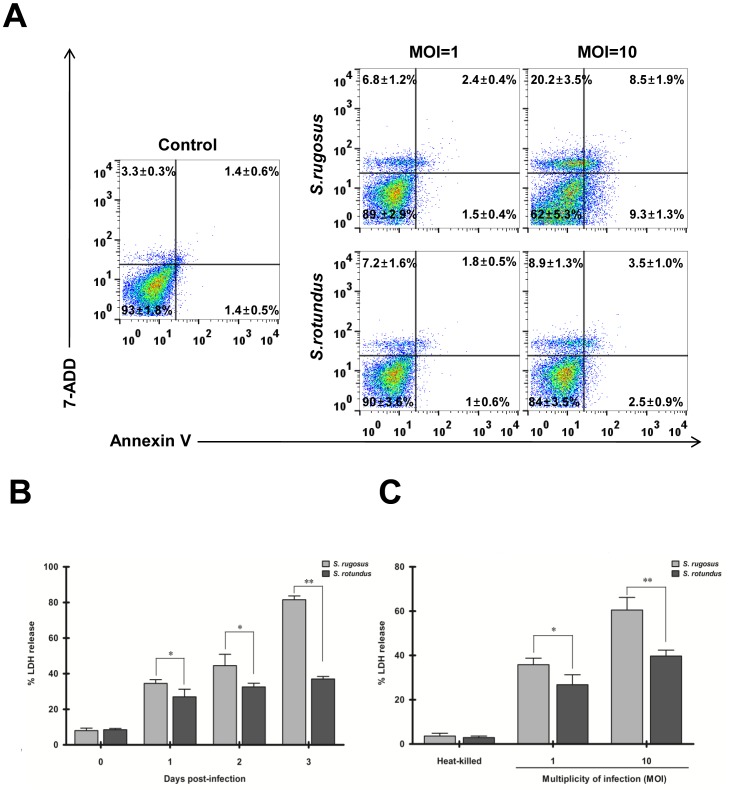
Quantitative analysis of necrosis and apoptosis in BMDMs infected with *S. rugosus* or *S. rotundus.* A) Apoptotic and necrotic cell death measured by 7-ADD and Annexin V assay. BMDMs were infected with *S. rugosus* or *S. rotundus* at a MOI of 1 or 10 for 24 h. After incubation, cells were collected and Annexin V-PE and 7-AAD were added. Samples were analyzed using flow cytometry. Results represent mean percentage of positive cells ± SD of all experiments performed. This experiment was repeated three times with similar results. B) LDH released from BMDMs infected with *S. rugosus* and *S. rotundus* at MOI of 1 according to the days post-infection. C) LDH released from BMDMs infected with *S. rugosus* and *S. rotundus* at MOI of 1 or 10 for 24 h. Culture supernatants were harvested and the release of LDH was assessed using a cytotoxicity detection kit. This experiment was repeated three times with similar results (*p<0.05 or **p<0.01).

Cell death caused by infection with intracellular pathogens can occur in many types of cells, including macrophages [22]. To determine whether this cytotoxic effect differs between the two *Segniliparus* spp., cell death was assessed using Annexin V/7-AAD and lactate dehydrogenase (LDH) assays. Exposure to the appropriate fluorescent dye (Annexin V for apoptosis and 7-AAD for loss of cell membrane integrity) allowed us to distinguish between viable, apoptotic, and early and late necrotic cells. Both species induced similar levels of necrotic macrophage cell death at a MOI of 1, but at a MOI of 10 a significant increase in cell death, including apoptosis, early necrosis, and late necrosis, was seen in BMDMs infected with *S. rugosus* compared to BMDMs infected with *S. rotundus* (Fig. 3A). These results indicate that *S. rugosus* is more cytotoxic to macrophages than *S. rotundus*. Additionally, the LDH assay showed that *S. rugosus* induced significantly greater loss of cell membrane integrity in a time-dependent (Fig. 3B) and a MOI-dependent manner (Fig. 3C) than *S. rotundus*. Heat-killed bacteria of both species did not exhibit any cytotoxic effects on macrophages, which suggest that only live bacteria induce cytotoxicity in macrophages (Fig. 3C).

### *Segniliparus* spp.-induced Cytokine Production from Macrophages is Mediated by TLR2 Signaling

**Figure 4:**
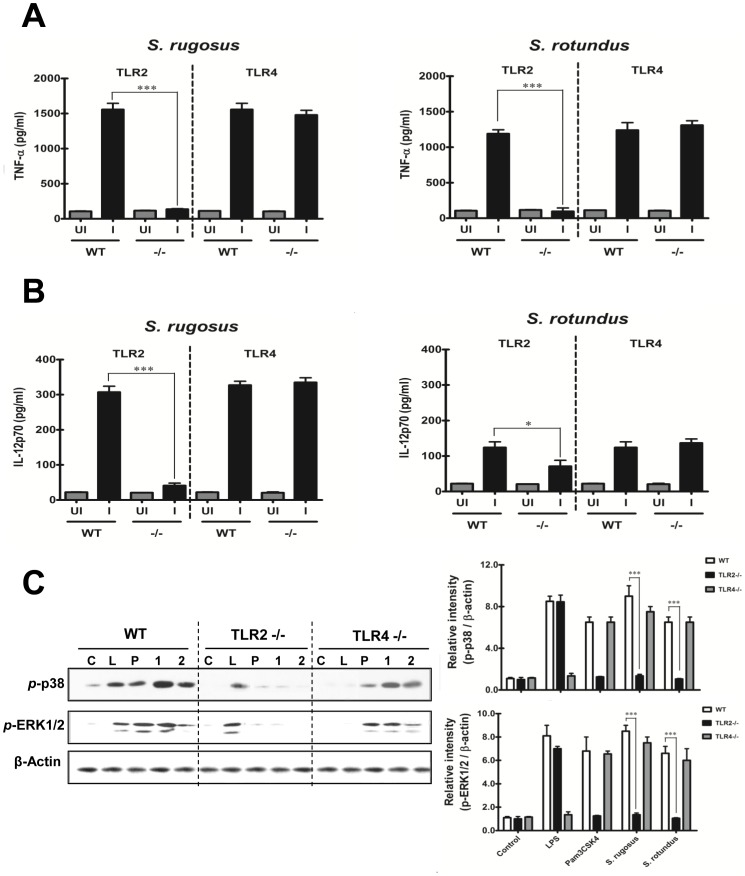
TLR2 plays an essential role in *S. rugosus* and *S. rotundus*-induced inflammatory cytokine production by BMDMs. A, B) BMDMs from wild-type, TLR2^−/−^ and TLR4^−/−^ mice were infected with *S. rugosus* and *S. rotundus* for 24 h at a MOI of 1 and then screened for the secretion of cytokines by enzyme-linked immunosorbent assay (A: TNF-α and B: IL-12p70). A representative experiment of three independent replicates with similar results is shown. C) MAPK activation was detected by Western blotting using lysates of cells infected with *S. rugosus* or *S. rotundus* for 1 h at a MOI of 10, or uninfected BMDMs, to detect activated ERK1/2 and p38 using phospho-specific antibodies as described in “Materials and Methods”. The same blots were washed and re-probed for β-actin as a loading control. A representative experiment of three independent replicates with similar results is shown. The related intensities of the phospho-MAPK band were analyzed by densitometry, and all densitometry values were normalized to the β-actin protein. The densitometry values are depicted as the mean ± SD of three independent experiments (*p<0.05 or ***p<0.001). C: uninfected; L: LPS-stimulated; P: Pam3CSK-stimulated; 1: *S. rugosus*-infected; 2: *S. rotundus*-infected.

To investigate the cellular signaling in response to the *Segniliparus* spp., BMDM cultures from TLR2- and TLR4-deficient and wild-type (WT) C57BL/6 mice were established and infected with either *S. rugosus* or *S. rotundus* at a MOI of 1. This allowed us to investigate the MAPK signaling pathway because it is activated by engagement of TLRs. The stimulation of murine TLR2-deficient macrophages returned *Segniliparus* spp.-induced TNF-α and IL-12p70 production to basal levels, indicating a critical role for TLR2 in *Segniliparus* spp.-induced cytokine production in macrophages (Fig. 4A and 4B). In addition, p38 phosphorylation and ERK phosphorylation was dependent on TLR2 signaling (Fig. 4C).

### Kinetic Analysis of p38 Phosphorylation, ERK Phosphorylation, and NF-κB Activation Shows Differences between *S. rugosus* and *S. rotundus*

**Figure 5:**
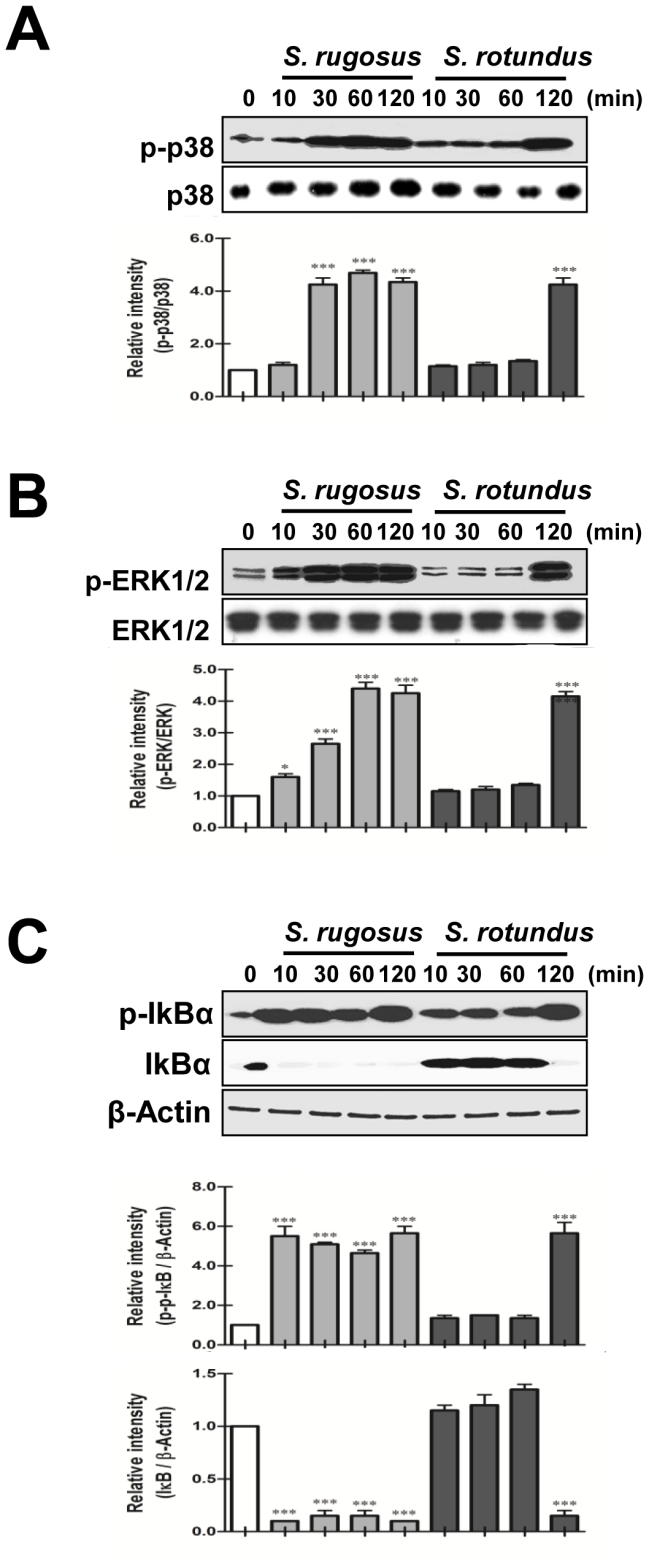
*S. rugosus* and *S. rotundus* induced activation of MAPKs and NF-κB. A–C) BMDMs were infected with *S. rugosus* or *S. rotundus* at a MOI of 10 and protein expression is shown at the indicated times. Cell lysates were subjected to SDS-PAGE, and immunoblot analysis was performed using specific antibodies to phospho-p38 (p-p38), p38, phospho-ERK1/2 (p-ERK1/2), ERK1/2, phospho-IκB-α (p-IκB-α), and IκB-α. Relative band intensities of each protein are expressed as a percentage compared to the value of untreated controls. The results shown are typical of three experiments for each condition. The data are shown as mean ± SD and statistical significance (*p<0.05 or ***p<0.001) is indicated for cells infected with *S. rugosus* versus *S. rotundus*.

To investigate the reason for the different levels of cytokine production in macrophages in response to *S. rugosus* and *S. rotundus*, we analyzed p38 and ERK MAPK activation in macrophages after *in vitro Segniliparus* spp. infection at a MOI of one. p38 and ERK phosphorylation was evaluated by immunoblot assay in macrophages infected with either *S. rugosus* or *S. rotundus* with cell lysates harvested at 0, 10, 30, 60, and 120 minutes. Interestingly, *S. rugosus* rapidly triggered p38 phosphorylation at 30 min and ERK MAPK phosphorylation at 10 min, and this phosphorylation persisted through to 120 minutes. In contrast, *S. rotundus* had only just begun to trigger both MAPKs at 120 min (Fig. 5A and 5B). Because many intracellular pathogens are known to trigger the NF-κB pathway, which is also involved in macrophage activation, we performed immunoblot assays to compare the ability of *S. rugosus* and *S. rotundus* to induce NF-κB activation in BMDMs (Fig. 5C). We found that *S. rugosus* rapidly induced the phosphorylation and degradation of IkBα at 10 min post-infection, but *S. rotundus* only showed induction of phosphorylation and degradation of IkBα at 120 minutes. These experimental data suggest that *S. rugosus* induces MAPK and NF-κB activation in BMDMs significantly faster and to a greater extent than *S. rotundus* after infection.

### *S. rugosus* causes Severe Pathology with a High Bacterial Colonization

**Figure 6:**
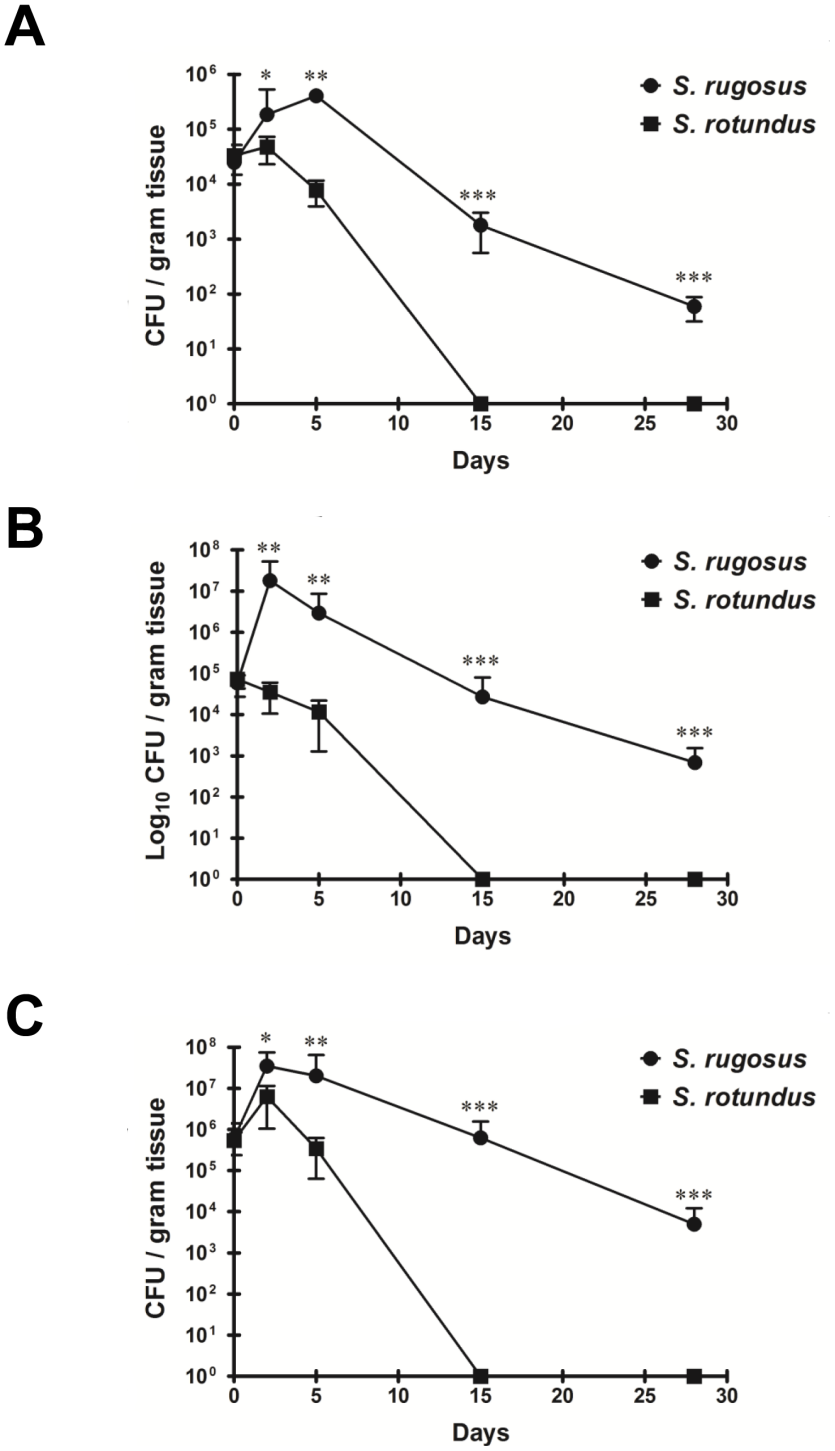
Growth profiles of *S. rugosus* and *S. rotundus* in C57BL/6 mice. C57BL/6 mice were injected intravenously with 10^7^ CFU of *S. rugosus* and *S. rotundus*, and the bacterial load of their lungs, spleens, and livers (A, B and C, respectively) were determined at 2, 5, 15, and 28 days post-infection. The data show the mean ± SD bacterial loads of five to six mice per group per time point experiments (**p<0.01 or ***p<0.001). The results represent one of three independent.

**Figure 7:**
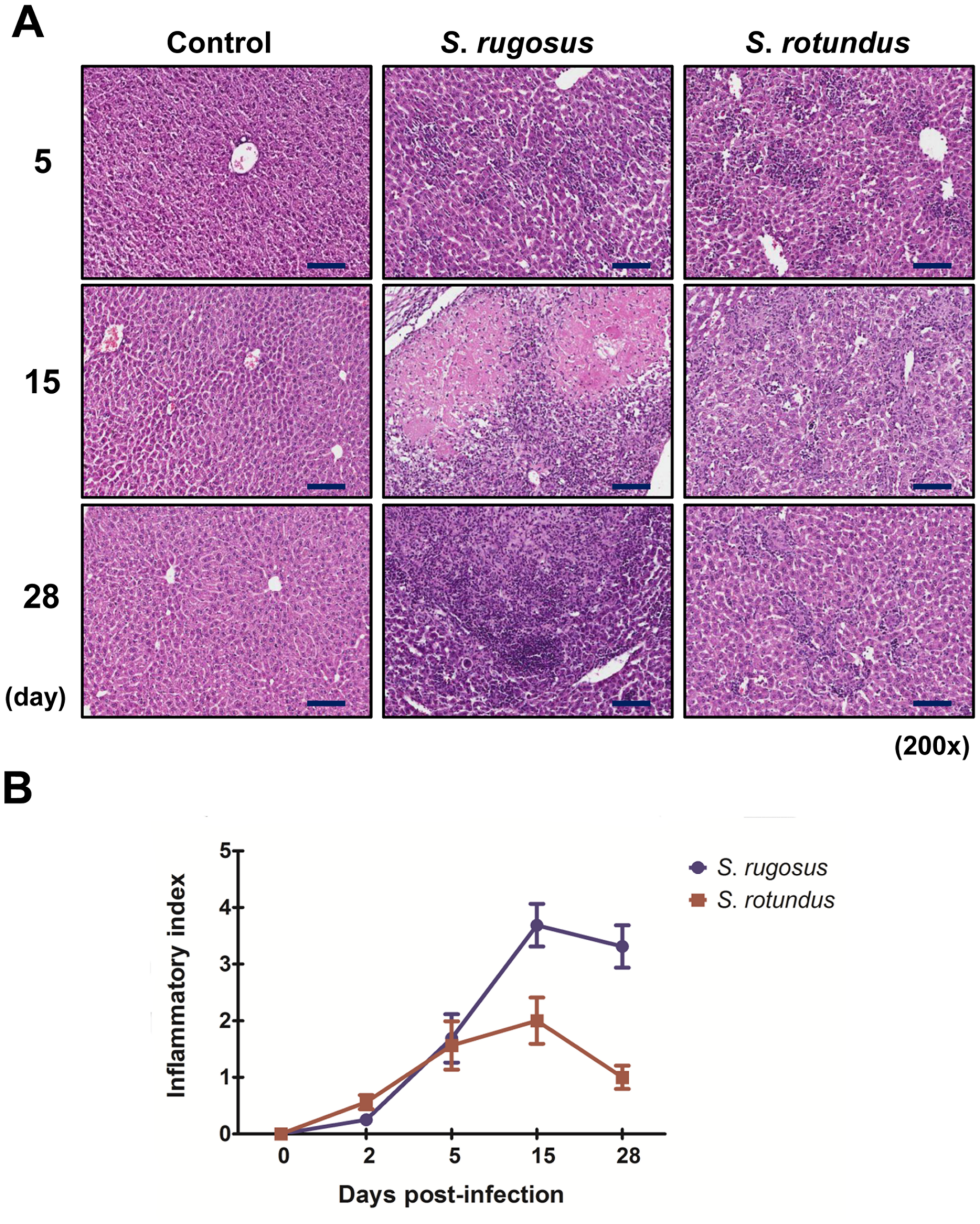
Histopathological analysis of liver infected with *S. rotundus* and *S. rugosus*. A) Histopathology of mice liver infected with *Segniliparus* spp. At 5, 15, and 28 days post-infection, mice were sacrificed and liver sections were stained with hematoxylin and eosin (bar, 100 µm). B) Inflammatory scores of hematoxylin and eosin-stained sections of liver. The line graph denotes the average of inflammatory score observed in livers following infection with *S. rotundus* and *S. rugosus* (four mice per group per time point experiments). Each section was blindly evaluated and scored as: absent (0), mild (1), moderate (2), severe (3) and extensively severe (4). This histopathological evaluation was made by a specialized liver pathologist.

**Figure 8:**
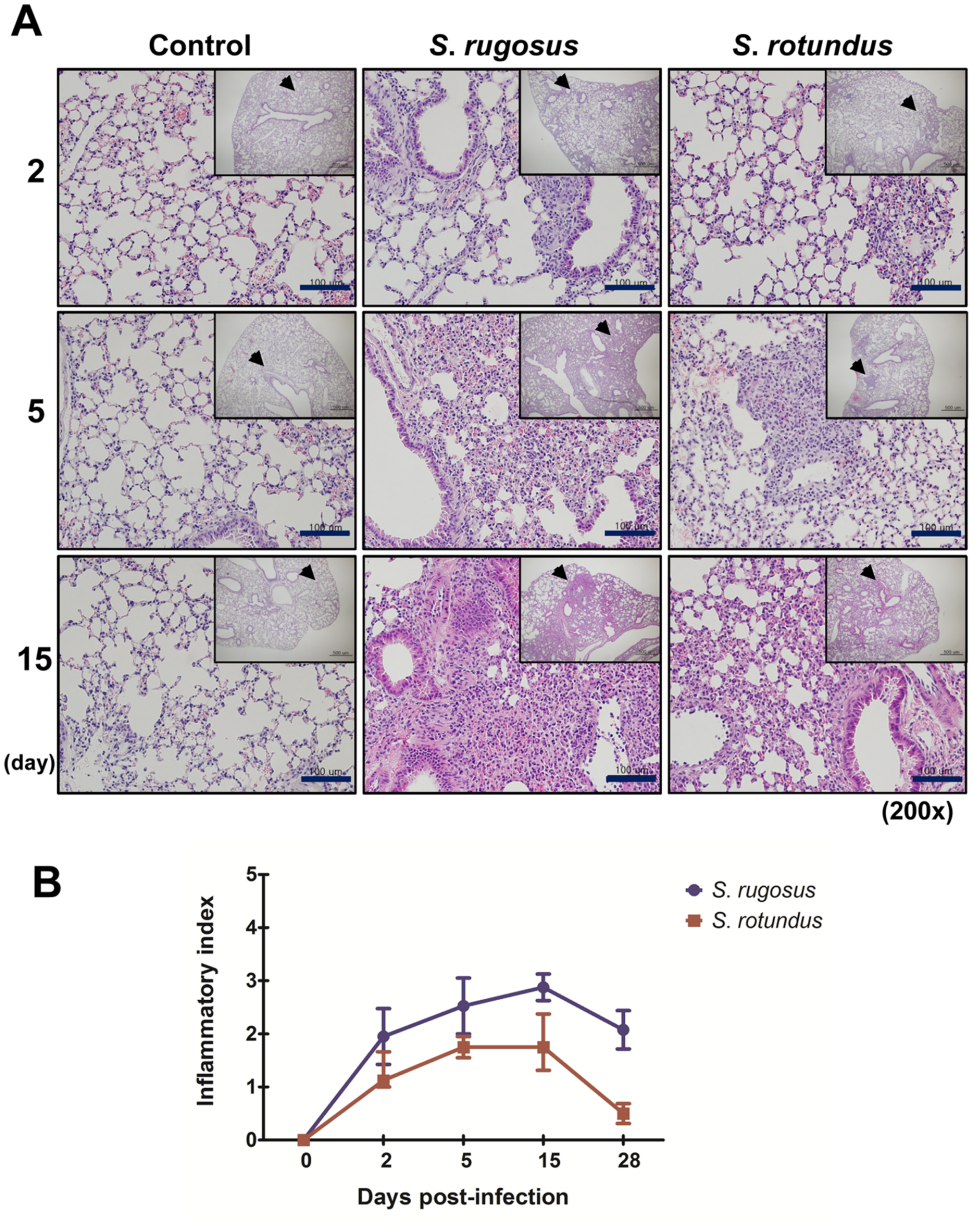
Histopathological analysis of lung infected with *S. rotundus* and *S. rugosus*. A) Histopathology of mice lung infected with *Segniliparus* spp. At 2, 5, and 15 days post-infection, mice were sacrificed and lung sections were stained with hematoxylin and eosin (bar, 100 µm). B) Inflammatory scores of hematoxylin and eosin-stained sections of liver. The line graph denotes the average of inflammatory score observed in lungs following infection with *S. rotundus* and *S. rugosus* (four mice per group per time point experiments). Each section was blindly evaluated and scored as: absent (0), mild (1), moderate (2), severe (3) and extensively severe (4). This histopathological evaluation was made by a specialized lung pathologist.

To compare the *in vivo* pathogenicity between *S. rugosus* and *S. rotundus*, we infected mice with intravenous injections of 1×10^7^ CFU of one of the two species. Mice infected with *S. rotundus* showed no significant increase in bacterial CFU, whereas those infected with *S. rugosus* showed a relatively large increase in the number of bacterial CFU in all organs tested at 5 days post-infection (Fig. 6). In addition, the increase in CFU counts was maintained through 28 days post-infection in mice infected with *S. rugosus*, but the *S. rotundus* infection showed a dramatic decrease and no CFUs were recovered from the lungs at 15 days post-infection (Fig. 6). Although C57BL/6 mice developed an established infection in the lungs after intravenous infection with *Segniliparus* spp., liver sections showed more obvious response to *Segniliparus* spp. infection than lungs (Fig. 7 and 8). More time-dependent inflammation was observed in livers and lungs by 15 days post-infection regardless of the *Segniliparus* spp. (Fig. 7 and 8). No definitive formation of granulomas was seen in mice infected with *S. rotundus* in both lungs and livers during infection. Only minor lymphocytic inflammatory responses were seen in these mice while most animals infected with the *S*. *rugosus* displayed primarily active immune responses with lymphocytic inflammation and granuloma-like inflammations in the lungs from the early stage of infection (2−5 days post-infection) (Fig. 7 and Fig. 8). However, significant granuloma formations were typically observed in the livers from mice infected with *S. rugosus* only in later stages of infection (15−28 days post-infection) (Fig. 7). These multifocal granulomatous inflammations consist of aggregation of macrophages surrounded by lymphocytes occasionally accompanied by thin layers of fibrous connective tissues and hepatocellular death (Fig. 7A). In addition, peri-bronchiolitis was observed in the lungs from mice infected with *S. rugosus* only over 15 days post-infection (Fig. 8). For histopathological scoring analysis, two independent pathologists examined the tissue section based on the size and number of inflammatory foci or granulomas. The severity of inflammation reached a score 3–4 in the livers and 2.5–3 in the lungs out of 4 on 15 days post-infection for mice subject to *S. rugosus* infection. In contrast, the inflammatory response of mice infected with *S. rotundus* ranged between the levels of 1 and 2.5 in both the lungs and livers during infection (Fig. 7B and Fig. 8B).

### Profiles of Pulmonary Cytokines and Infiltrating Immune Cells

**Figure 9:**
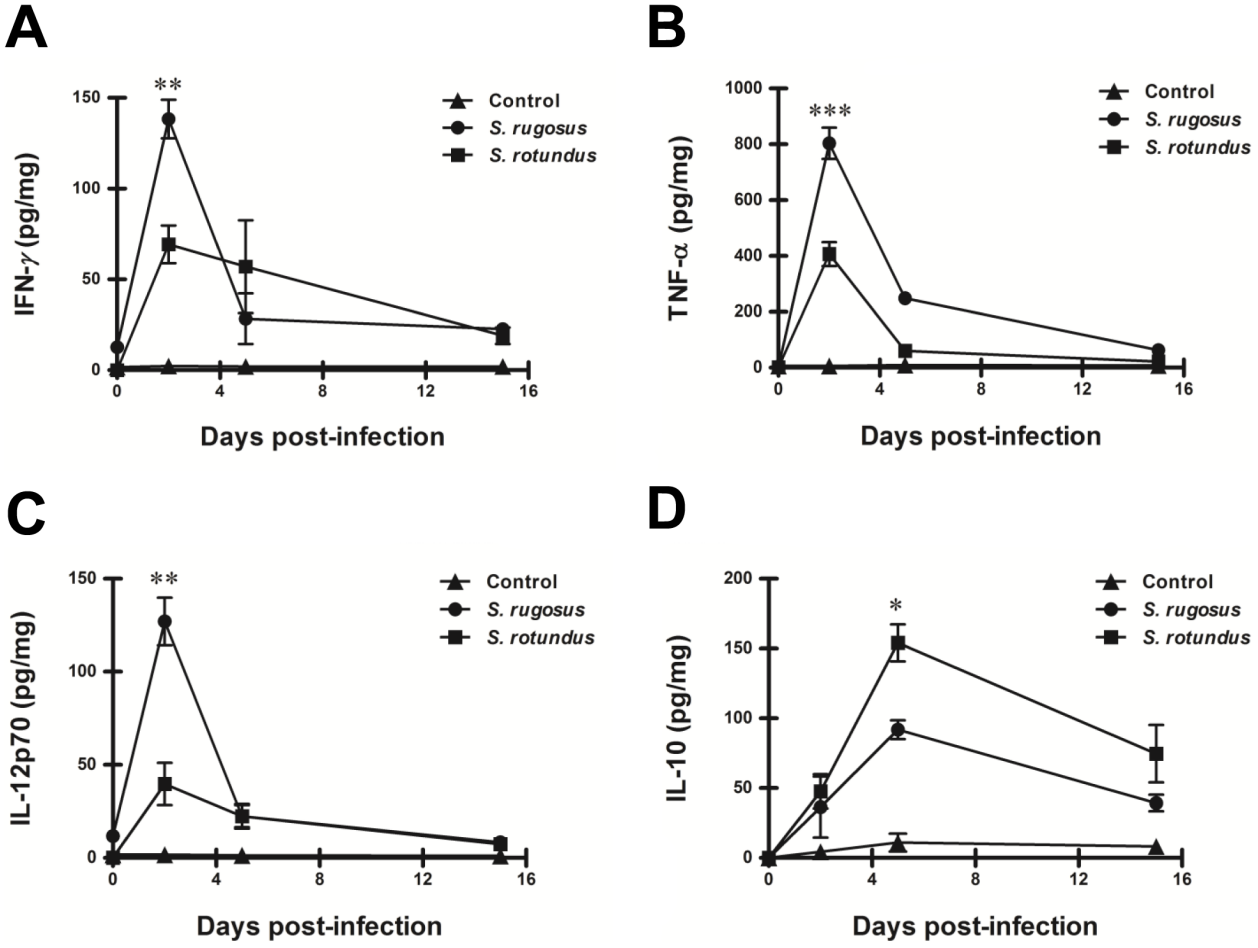
BAL fluid cytokine response levels in C57BL/6 mice infected with *S. rugosus* and *S. rotundus.* BAL was performed after infection at the times indicated, and the BAL fluids were analyzed for (A) IFN-γ, (B) TNF-α, (C) IL-12p70, and (D) IL-10 levels as described in “Materials and Methods”. The results are presented as means ± SD of each group (*p<0.05, **p<0.01, or ***p<0.001). This experiment was repeated three times with similar results.

**Figure 10:**
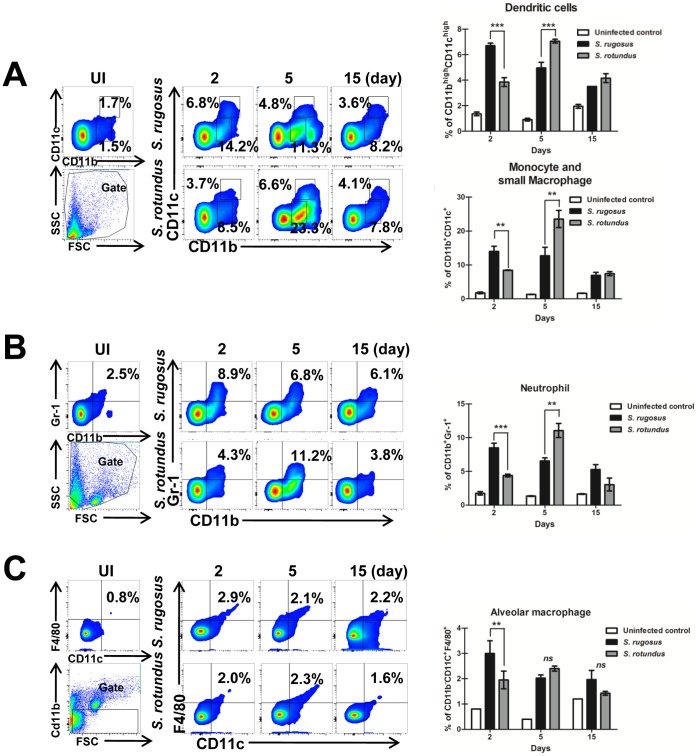
Infiltration of immune cells into the bronchoalveolar space in C57BL/6 mice infected with *S. rugosus* and *S. rotundus*. BAL cells isolated from mice infected with *S. rugosus* and *S. rotundus* at day 2, 5 and 15 post-infection were stained with the indicated antibodies and were analyzed by flow cytometry. A) CD11c^high^CD11b^high^ cells indicate dendritic cells. CD11c^int^CD11b^+^ cells indicate small macrophage and monocyte. B) CD11b^+^Gr-1^+^ cells indicate neutrophils. C) CD11b^−^CD11c^+^F4/80^+^ cells indicate alveolar macrophages. The bar graph represents the percentages of cells in the quadrants or squares. The results are presented as means ± SD of each group (**p<0.01, or ***p<0.001).

Cytokine production of IFN-γ, TNF-α, IL-12p70, and IL-10 were measured in bronchoalveolar lavage [18] samples and compared between mice infected with the two species. The production of IFN-γ, TNF-α, and IL-12p70, increased to a statistically significant level in the *S. rugosus*-infected mice compared with *S. rotundus*-infected mice at 5 days post-infection (Fig. 9A–C). In contrast, the anti-inflammatory cytokine IL-10 significantly increased in the *S. rotundus*-infected mice (Fig. 9D). Furthermore, we analyzed BAL cells to determine the type of infiltrating immune cells into the bronchoalveolar space. Interestingly, infiltration of CD11b^high^CD11c^high^ dendritic cells, CD11b^+^CD11c^int^ monocytes and small macrophages, CD11b^+^Gr-1^+^ neutrophils and CD11b^-^CD11c^+^F4/80^+^ alveolar macrophages into the bronchoalveolar space increased markedly in mice infected with *S. rugosus* at 2 days post-infection while these cells presented the highest recruitment at 5 days post-infection in *S. rotundus*-infected mice (Fig. 10).

### Humoral and Cellular Immune Responses to *Segniliparus* spp. during Infection

**Figure 11:**
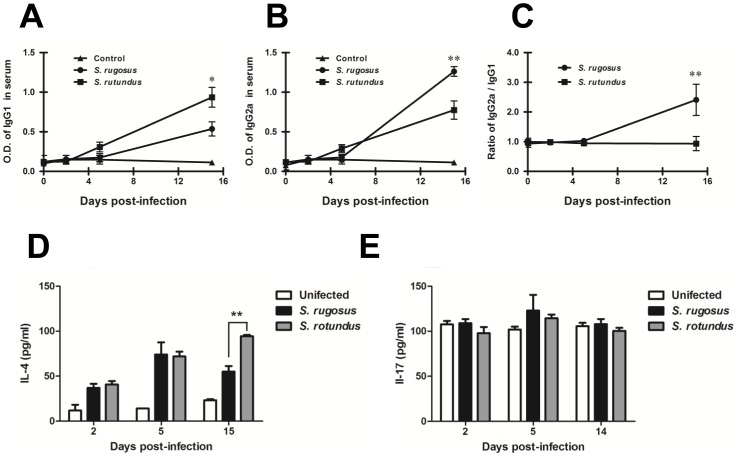
Levels of humoral responses in C57BL/6 mice following infection with *S. rugosus* and *S. rotundus* at designated times. Antibody responses of IgG1 (A), IgG_2a_ (B) and the IgG_2a_/IgG1 ratio (C), IL-4 (D), and IL-17 (E) were measured by ELISA as described in “Materials and Methods”. Each sample was examined in triplcate. Data are presented as the absorbance at 450 nm and are representative of three experiments. The results are presented as means ± SD of each group (*n* = 6 per group, *p<0.05, **p<0.01).

**Figure 12:**
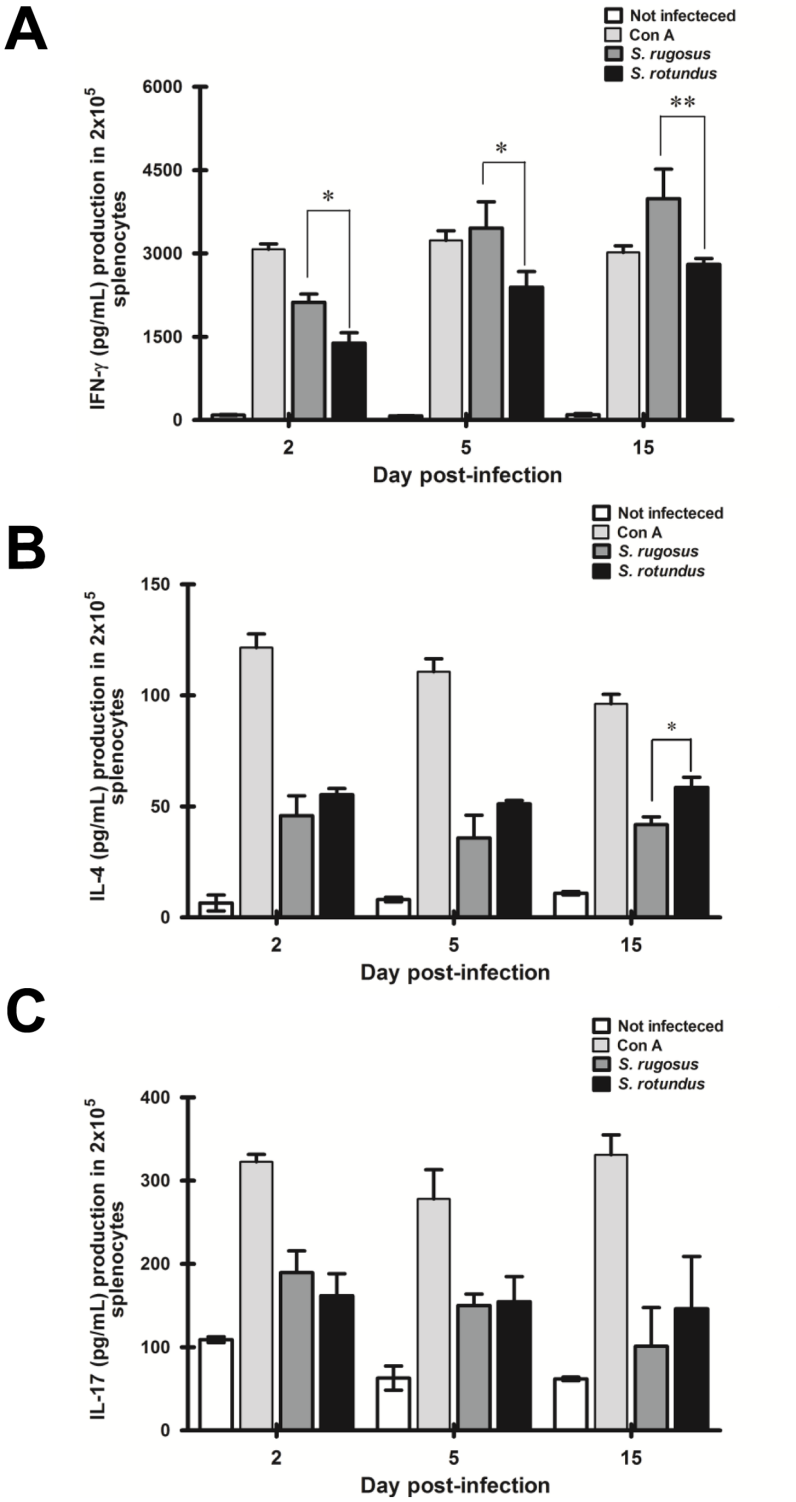
Levels of cellular immune responses in C57BL/6 mice following infection with *S. rugosus* and *S. rotundus* at designated times. Levels of IFN-γ (A), IL-17 (B) and IL-4 (C) produced by splenocyte cultures upon re-stimulation with *S. rugosus* and *S. rotundus* bacterial lysates were evaluated by sandwich ELISA of culture supernatants. Concanavalin A (ConA) was used as a positive control (2.5 µg/mL), and medium alone was used as a negative control. The results are presented as means ± SD of each group (*n* = 6 per group, *p<0.05, **p<0.01).

Antibody responses induced by *Segniliparus* spp. infection were compared after intravenous infections according to their respective cellular extract antigens and time periods. An IgG1 antibody response was induced in mice infected with *S. rotundus* (Fig. 11A), but the *S. rugosus* infection resulted in an IgG_2a_ antibody response (Fig. 11B). Interestingly, the ratio of IgG_2a_ to IgG1 only increased significantly in mice infected with *S. rugosus* at 15 days post-infection (Fig. 11C). In addition, the level of IL-4 increased in serum from mice infected with *S. rotundus* at 15 days post-infection (Fig. 11D) while no significant changes were detected in IL-17 level during infection regardless of *Segniliparus* spp. (Fig. 11E). We next investigated the ability of the two species to elicit cell-mediated immune responses in spleen following by the infection. Overall, similar time-dependent patterns of IFN-γ production were observed in both infections, but a greater production of IFN-γ was detected in response to *S. rugosus* than in response to *S. rotundus* (Fig. 12A). In contrast, a higher production of IL-4 in response to *S. rotundus* than in response to *S. rugosus* was observed only at days 15 post-infection (Fig. 12B). The different levels of IL-17 were not observed between both infections (Fig. 12C). Constant production of IFN-γ, IL-4 and IL-17 were observed upon stimulation with Concanavalin A.

## Discussion

Although the *Segniliparus* spp. are recognized as emerging pulmonary pathogens in humans, very little is known about their pathophysiological features. It is not even known if they are intracellular or extracellular pathogens. As a first step toward characterizing the basis for the pathogenicity of *S. rugosus* and *S. rotundus*, we have identified phenotypic differences between the two species in macrophage and mouse models of infection.

This study demonstrates for the first time fundamental differences in the innate immune responses, signaling pathways, virulence, and pathogenicity between *S*. *rugosus* and *S*. *rotundus*. The virulence of intracellular pathogens can be determined by quantifying the number of cells in the infection, measuring the cytotoxic effects in the targeted cells, and assessing the ability of the organism to persist and produce pathological reactions at the infected sites in animals. We were able to conclude that *S*. *rugosus* is overall more virulent than *S*. *rotundus* by comparing intracellular growth, levels of induction of pro-inflammatory cytokines, cytotoxicity, histopathology, and the *in vivo* colonization and persistence of the two species.

The two *Segniliparus* spp. resemble rapidly growing mycobacteria such as *Mycobacterium abscessus* and *M. smegmatis* with respect to their *in vitro* growth pattern, acid-fastness, biochemical features, morphotype variation, production of mycolates, *in vivo* granuloma formation, and signaling pathways [23]. Previous studies of *M. abscessus* colony morphotypes showed that the rough variant had different glycopeptidolipids (GPLs) or lacked them altogether, and induced a greater inflammatory response than the smooth morphotype [13], [23]. This indicated that *M. abscessus* with the rough morphotype is more pathogenic and invasive. In a similar manner, *S. rugosus* grew as wrinkled and rough colonies but *S. rotundus* grew as smooth, dome-shaped colonies on Middlebrook 7H10 agar. Our study showed that *S*. *rugosus* is more virulent than *S*. *rotundus* by showing that *S. rugosus* is more persistent and rapidly replicates inside macrophages and results in a higher level of pro-inflammatory cytokine production and cell death. These results were also verified in *in vivo* infection models.

*S. rotundus* and *S. rugosus* produced mycolic acids varying over a much wider mass range, and lacked oxygen containing R groups, as compared to *Mycobacterium* spp. [4]. In normal phase, one-dimensional thin layer chromatography analysis, three separate mycolic acid methyl ester (MAME) bands were seen in *S. rotundus* and likely correspond to long, medium, and short chain mycolates. *S. rugosus*, on the other hand, produced only two spots, likely corresponding to medium and long chain MAMEs [4]. The precise role for such a structural diversity of *Segniliparus* mycolic acids is unknown, but these differences probably influence host immune responses as well as pathogenesis. Further studies are required to understand how the unique structural properties of the mycolic acid components between the two species influence host-pathogen interactions.

Although the virulence of intracellular pathogens is associated with their ability to persist and replicate in macrophages after infection, there is no definitive evidence as to whether or not the genus *Segniliparus* are intracellular pathogens. Thus, we first investigated whether the two *Segniliparus* spp. could multiply in macrophages and in epithelial cells. In contrast to macrophages, epithelial cells maintained non-replicating states of the two *Segniliparus* spp. at a MOI of 1 for 5 days, indicating that macrophages may be the primary target cells of these pathogens. It is well known that virulent strains of *Mycobacterium* spp. grow more rapidly than avirulent or attenuated strains in phagocytes [13], [24], [25]. In the present study, the type strain *S*. *rugosus* multiplied faster in macrophages than *S*. *rotundus*. We also found that *S*. *rugosus*, but *S*. *rotundus*, was cytotoxic to murine BMDMs, suggesting that a cytotoxic effect plays a major role in the virulence of *S*. *rugosus* during infection. *S*. *rugosus* also caused increased pro-inflammatory cytokine release, and heat-killing the bacteria abrogated these enhanced levels. In general, it is well documented that lipoproteins produced by many bacteria trigger immune responses via TLR2 [26], [27], [28]. Thus, it is believed that this abrogation of immune responses by heat-killed *Segniliparus* spp. was resulted from the denaturation of TLR2-stimulating lipoproteins.

Macrophages infected with pathogenic intracellular pathogens undergo both apoptosis and necrosis [29], [30]. In general, apoptosis is associated with antimicrobial activity, but necrosis results in the spread of infection in conjunction with host cell lysis and the subsequent destruction of surrounding tissues [30]. It has been well documented that the ability to induce necrosis is associated with the virulence of intracellular pathogens [30], [31]. In this study, we observed that *S. rugosus* induced more macrophage cell death, and of a more necrotic (less apoptotic) phenotype, suggesting that cytotoxic effects may play an important role in the virulence of *S. rugosus* during infection.

TNF-α, a crucial pro-inflammatory cytokine, is essential for the formation of the anti-intracellular bacterial immune response, and regulates the expression of other cytokines that contribute to a protective immune response [32]. The production of IL-12 is essential for the development of host Th1 immunity and resistance against intracellular pathogens [33]. IFN-γ is a central cytokine that inhibits intracellular pathogens as demonstrated by the high susceptibility of mice with a defective IFN-γ gene to mycobacterial infection and salmonellosis [34], [35]. Increases in the number of IFN-γ-secreting cells were positively correlated with decreased bacterial load at the infected sites [35], [36]. The infiltration of immune cells and expression of cytokines are critically associated with the severity of disease states in infections with intracellular pathogens [37].

Thus, cytokine profiles upon *Segniliparus* spp. infection were studied and compared in *ex vivo* and *in vivo* models of infection. Macrophages, as the primary innate cells, secrete various kinds of cytokines in response to intracellular pathogens, and both *Segniliparus* reference strains were potent inducers of cytokine secretion from macrophages in a MOI-dependent manner. This property was somewhat specific to the species; TNF-α, IL-12p70 and IL-6 were up-regulated upon *S. rugosus* infection, and IL-10 was upregulated upon *S. rotundus* infection.

The different abilities of the two *Segniliparus* spp. to induce the production of pro-inflammatory cytokines such as TNF-α and IL-12p70 seems to be directly related to their virulence as assessed in murine models and macrophages. In addition, the present study has shown a positive association between progressive inflammation and the amount of IFN-γ production observed in mice infected with *S. rugosus*. The protective response to intracellular pathogens is complex and multifaceted involving many components of the immune system. In particular, antigen-presenting cells such as macrophages and DCs play key roles in host immune response to infections [38]. Furthermore, lung infection by pathogenic bacteria leads to the massive recruitment of neutrophils into infected airways. A primary function of recruited neutrophils is pathogen elimination. Neutrophils play a primary and unambiguous role in pathogenic bacteria clearance during acute pulmonary infection [39], [40]. These cells have ability to secrete TNF-α, IL-12p70, and IL-6 and can be infiltrated more when infected with pathogenic bacteria [39], [41]. In addition, these cytokines have a variety of immunoregulatory effects, which may influence bacterial killing and generate inflammation. In the present study, neutrophils, DCs, and macrophages, the main inflammatory cells for innate immunity, were infiltrated rapidly into the lung in *S. rugosus* infection, indicating that the host may require more vigorous immune responses to eliminate *S. rugosus* than *S. rotundus*.

Many pathogens trigger signaling pathways, such as MAPK and NF-κB, that are involved in cytokine response and inflammation [11]. These responses are linked to the association between pattern recognition receptors (PRRs) and pathogen-associated molecular patterns (PAMPs) as has been demonstrated for other intracellular pathogens [11]. However, very little information is available about any PAMPs that might be associated with *Segniliparus* infection. Among various PRRs, TLRs are a family of membrane proteins that play important roles in detecting microbes and triggering innate immune responses upon pathogen recognition. Recognition of microbial components by TLRs plays a central role in the immune system’s decision whether to respond to a particular microbial infection. Activation of signaling through TLRs results in recruitment of the adaptor molecule MyD88 and ultimately leads to activation of MAPKs and translocation of NF-κB to the nucleus [12].

TLR2 signaling is reportedly required for the control of intracellular pathogens such as *Mycobacterium* spp [42], [43]. MAPKs, which are triggered by pathogens through engagement of TLRs, are important signal-transducing enzymes involved in many facets of cellular regulation and cytokine responses [10], [11], [12]. Such signaling pathways were also activated in the *Segniliparus* spp. in a similar manner to signaling pathway activation in rapidly growing mycobacteria [44], [45].

Both *S. rugosus* and *S. rotundus* induce NF-κB nuclear translocation and activate the p38 and ERK MAPK signaling pathways via TLR2 signaling. Previous studies have indicated that p38 and ERK activation are required for mycobacteria-induced TNF-α secretion [45], [46]. *S. rugosus* triggered faster and greater activation of MAPK signaling and IκB degradation than *S. rotundus*, indicating that *S. rugosus* rapidly signals macrophages through MAPK and NF-κB to trigger the up-regulation of pro-inflammatory cytokines. The initiation of the cytokine response induced by *S. rugosus* in macrophages begins within minutes after infection and a difference between the two species is seen already at very early time points. This *in vitro* effect may be reflective of the inflammation and clinical significance of *S. rugosus*.

The MAPK family is composed of three major serine-threonine protein kinases including p38, ERK and the c-Jun NH2-terminal kinase [47] Several studies have compared MAPK signaling in macrophages during various mycobacterial infections, and these suggest that the degree and timing of p38 and ERK1/2 activation differ in response to different mycobacterial organisms. For example, early signal transduction events induced by different strains or closely related species also showed differential induction of cytokines [45], [46]. Such differential requirements for the activity of both pathways for TNF-α and IL-10 release could be related to bacterial virulence. Several studies have shown that the differential activation of MAPKs in terms of timing and degree are correlated with differences in colony morphotypes among genetically similar bacteria. For example, *M. abscessus* colonies often display a rough morphotype as a result of a deficiency of GPL on the bacterial surface, and these strains are more pathogenic, activate stronger MAPK signal transduction, and result in increased inflammatory response compared to the smooth-colony morphotype [46], [48]. Interestingly, *S*. *rugosus* has an irregular, wrinkled, and rough morphotype whereas *S*. *rotundus* has a round and smooth morphotype.

In general, TLR2 is known to recognize lipidated antigens, including mycobacterial glycolipids such as phosphatidyl-(myo)-inositol mannosides, lipomannans, lipoarabinomannans, and inositol phosphate-capped lipoarabinomannans [44], [49]. Thus, the different immune responses and the degree of MAPK activation between *S. rugosus* and *S. rotundus* are likely due to the diversity of lipids in the outer layer of the bacterial cell wall, including mycolic acid. Investigations into the differences among the lipids on the surface of the *Segniliparus* cells will be required to elucidate the precise mechanism of signal activation.

This study has several limitations. First, this study was conducted using a single type strain of each species; therefore, it may be hard to generalize the main conclusion of this study. To determine whether these findings are clinically significant, the further studies using more clinical isolates are necessary. Second, the natural infection course of *Segniliparus* spp. may be differed from our infection model. According to previous reports [3], [6], airborne infection is thought to be the primary route of *Segniliparus* spp. infection. However, we employed an intravenous infection model with a high infectious-dose. Thus, the bacterial pathogenesis and immunological events could not be similar to those of human infections. Although the route of bacterial infection is extremely important, an intravenous infection has several advantages when comparing similar pathogens in aspects of their unknown pathogenicities or the host immune responses to them. It is advantageous i) to equate the infectious dose because the bacterial distribution in the organs and the host immune responses are largely influenced by initial inoculum size [50], ii) to compare the bacterial persistence and disease progression for a long-term period, iii) to evaluate pathological reactions in multiple organs, and iv) to investigate active and systemic immune responses, elicited rapidly. Third, this study was preliminary because we did not identify the species-specific virulent factors of two species. Despite several limitations, our study demonstrated the phenotypic differences in the pathogenesis and immune responses of *S*. *rugosus* and *S*. *rotundus* infections.

In summary, *S*. *rugosus* is a more virulent species than *S*. *rotundus* in terms of intracellular growth rate, cell death induction, *in vivo* persistence, and immune responses. These results suggest why *S. rugosus* infections are diagnosed more frequently than *S. rotundus* infections. Further genetic investigation of the virulence factors specific to *S. rugosus* will provide pivotal clues for the determination of the disease characteristics and pathogenesis of *Segniliparus* spp.
